# Mental Health in Young Detainees Predicts Perpetration of and Desistance From Serious, Violent and Chronic Offending

**DOI:** 10.3389/fpsyt.2022.893460

**Published:** 2022-06-15

**Authors:** Steffen Barra, Daniel Turner, Petra Retz-Junginger, Priscilla Gregorio Hertz, Michael Rösler, Wolfgang Retz

**Affiliations:** ^1^Institute for Forensic Psychology and Psychiatry, Saarland University, Homburg, Germany; ^2^Department of Psychiatry and Psychotherapy, University Medical Center of the Johannes Gutenberg-University, Mainz, Germany

**Keywords:** psychiatric, disorder, juvenile offending, recidivism, incarceration, detention, delinquency

## Abstract

Mental health problems are common among young offenders but their role in predicting criminal recidivism is still not clear. Early identification and treatment of young offenders at risk of serious, violent, and chronic (SVC) offending is of major importance to increase their chances to develop into a healthy and non-criminal future and protect society from further crime. In the present study, we assessed mental health among 106 young offenders while incarcerated and analyzed their criminal careers up to 15 years after release. We found high rates of mental health issues, especially externalizing problems, but also concerning illegal substance and alcohol use patterns as well as personality disorders. Rule-breaking behavior and internalizing problems were negatively related to incarceration time until study assessment, but withdrawal and internalizing problems were positively associated with remaining time to release. Whereas, SVC status before assessment and after release were not statistically dependent, mental health issues predicted perpetration of and desistance from SVC offending after release. Alarming alcohol use appeared to be of specific importance in this regard. Findings indicate that young offenders at risk of future SVC offending may benefit from mental health treatment with specific focus on problematic alcohol consumption to prevent ongoing crime perpetration.

## Introduction

Two major aims of forensic psychiatry and psychology are (1) to assess and treat mental health issues in people at risk of criminal behavior and (2) to identify risk and protective factors that increase or reduce the risk of further delinquency. These aims become of specific importance when working with criminal adolescents or young adults because effective intervention may increase their chances to develop into a healthy adulthood desisting from future crime.

Mental health issues are common among young offenders. International studies have reported rates of psychiatric problems of up to 93% among young offenders, including externalizing–i.e., conduct disorder, oppositional defiant disorder, attention deficit hyperactivity disorder (ADHD)–but also internalizing (i.e., mood disorders, anxiety) problems, substance use disorders, and personality disorders (1–12). Rates differed with regard to the setting young people were assessed in, e.g., community-based treatment settings vs. incarceration, with the latter usually showing higher frequencies of mental health issues. However, it has been criticized that it often remains unclear to what extent mental health problems have existed before and thus may have led to criminal behavior, and to what extent placement circumstances may have influenced mental health [e.g., (1)].

There is a vast amount of research that has examined the predictive value of mental health problems in young offender samples with respect to future crime. In a recent meta-analysis including data of 5,737 juveniles, Wibbelink et al. (2) found small to moderate predictive effects of externalizing but not internalizing problems on criminal recidivism. Other studies reported similar results. Higher rates of criminal recidivism were found in young offenders with ADHD (3–5), conduct disorder (6), oppositional defiant disorder (7) as well as substance use disorders (8) and personality disorders [especially DSM Cluster B disorders (10)]. However, findings remain inconsistent across studies due to differences in definitions and assessment of mental health issues (e.g., self-reported vs. clinician-administered), recidivism (e.g., reconviction vs. reincarceration, self-reported vs. officially recorded), and crime concepts (e.g., in terms of severity and type of criminal acts). For example, mental health may relate differently to criminal recidivism when differentiating violent from non-violent crime. Bessler et al. (8), for instance, found that young offenders' mental health problems and substance use disorders in particular, were associated with risk of violent but not general (non-violent) criminal re-offending. Plattner et al. (10) concluded that substance use disorders were predictive of future non-violent, drug-related crime, but problematic alcohol use in particular was associated with violent criminal recidivism. Conversely, Mulder et al. (11) found a negative predictive relationship of psychopathology on violent reoffending. In addition, conclusive empirical evidence is lacking due to different follow-up periods among studies [e.g., adolescence vs. adulthood (11)].

However, considering differences in follow-up periods is of specific importance as delinquency has been claimed to be a common phenomenon during adolescence indicating that young people with repeated crime only during this developmental period may not be as burdened by mental health problems as those who continue criminal behaviors until adulthood (2, 12). A well-known perspective on young peoples' courses of delinquency is Moffit's developmental taxonomy on adolescent-limited and life-course-persistent antisocial behavior (13): According to this theory, most juveniles may engage in (non-pathological) antisocial behavior during “a contemporary maturity gap” (p. 674) but desist from crime after this period, whereas a smaller proportion showing early conduct problems and higher psychosocial burden continues repeated and more severe (pathological) criminal behaviors beyond adolescence. Moffitt (14) recently described evidence from 25 years of research on this taxonomy and emphasized that more research is needed, e.g., concerning associations of delinquent pathways with mental health.

Considering the developmental courses of delinquency as well as the potential individual and societal consequences, it appears of major importance to identify those young offenders who are at risk of serious, violent, and chronic (SVC) offending. SVC offenders have been suggested to be a rather small group but “responsible for a disproportionate amount of serious crime” (15). Following a cohort of more than 27.000 individuals over 16 years, Kempf-Leonard et al. (16) found that among young offenders with serious, violent, and chronic delinquency, those who had shown a combination of these three crime characteristics had the highest rates of adult crime perpetration. Baglivio et al. (17) examined the prevalence as well as risk and protective factors of SVC offending among more than 363.000 juveniles referred to the juvenile justice system in the US over a 5-year period. They reported a proportion of SVC offenders of 8.9%, with SVC status defined as having shown a history of four or more official referrals with at least one felony offense against a person or a weapon/firearm charge. Compared to non-SVC offenders, SVC offenders were younger at first referral and had more risk but less protective factors regarding criminal recidivism after 1 year follow-up. Although SVC offenders showed higher scores on history of mental health problems, current mental health did not differ between SVC and non-SVC offenders. Current substance use predicted future SVC rearrest. Among more than 64.000 young delinquents, Perez et al. (18) stated a proportion of 16.66% SVC offenders (defined as having committed three or more serious felony offenses with at least one violent offense) and found that a predictive effect of adverse childhood experiences (ACEs) on SVC offending was partially mediated by maladaptive personality traits (e.g., impulsivity) and adolescent problem behavior, including substance use and mental health problems.

In summary, the scientific foundation on the relations of mental health and SVC offending among young delinquents is still scarce. More long-term investigations are needed to shed light into the dynamics of mental health and other risk and protective factors with perpetration of and desistance from SVC offending in order to identify those young people at risk of continuous, severe crime involvement. Further empirical evidence may serve to elaborate adequate treatment and prevention approaches to increase young offenders' chances to develop into a healthy, crime-free adulthood and, thus, also contribute to the protection of society.

Considering abovementioned findings but also limitations of previous research, the present study aimed at examining the course of SVC offending among young detainees up to 15 years after release from incarceration and respective associations with mental health. We hypothesized that compared to SVC desisters, SVC offenders would show higher rates of mental health issues, especially externalizing problems. Baseline SVC status was suggested to be positively associated with future SVC status. We also expected that current externalizing problems, substance use problems and cluster B personality disorders would increase the risk of being a future SVC offender.

## Materials and Methods

### Procedure and Sample

Study procedures were described in detail in previous studies of our research group (5, 19–21). In short, baseline assessment took place at the Ottweiler Juvenile Detention Center in Saarland, Germany, between 2001 and 2002. In Germany, individuals cannot be legally arrested before they reach the criminal responsibility age of 14 years, and juvenile law is usually applied to offenders up to the age of 18 to 21 years. In Saarland, according to the enforcement plan of the state, juvenile sentences and pre-trial detention of male adolescents and young adults, who are under 21 years of age at time of the offense, are carried out in the Ottweiler Juvenile Detention Center. At the time of baseline data collection, of the *N* = 170 detainees who were initially asked to participate in the study, *n* = 41 (24.12%) refused to sign the informed consent form or had insufficient knowledge of the German language. Thus, after being informed about the study procedures and giving written consent (when detainees were younger than 18 years old, their legal caregivers provided informed consent), a total of 129 young male offenders were included in the study. At baseline, data on young offenders' biography, criminal history, and mental health were assessed by self-rating questionnaires and clinician-administered interviews conducted by trained psychologists and psychiatrists. In order to examine the young offenders' long-term criminal careers, we obtained official criminal records including any convictions in 2016, up to 15 years after release. In Germany, criminal records consist of convictions only, so they do not provide information about criminal charges. Of the initially 129 included young offenders, *n* = 21 could not be included in the follow up, as no criminal records were provided by justice authorities. Two more participants had to be excluded after combining the data sets (1 died, 1 could not be assigned). Subsequently, full follow-up information was available for 106 of the former 129 participants (5). Thus, we only considered their baseline and follow-up data for the present study. All participants had completely answered all included questionnaires. Thus, there were no missing data. Study procedures had been approved by the ethics committee of the medical chamber of Saarland, Germany.

Participants had been incarcerated at baseline assessment for the following index offenses: bodily harm *(n* = 30, 28.3%), sexual offending (*n* = 2, 1.9%), property related offenses (*n* = 38, 35.8%), narcotics related offenses (*n* = 12, 11.3%), homicide (*n* = 4, 3.8%), and arson (*n* = 1, 0.9%). On average, they were 18.33 years old when conducting the index offenses (*SD* = 1.77, range = 14–23 years). At baseline assessment, participants were 15–28 years old (*M* = 19.52, *SD* = 2.10). About half of the sample showed low educational levels (none or auxiliary school graduation compared to secondary school graduation or high school diploma; *n* = 56, 52.8%). For their index offense, participants had been incarcerated for 10.50 months on average at the time baseline assessment took place (*SD* = 9.72 months, range = 0.27–42.77 months) and they had to face a mean of 13.51 more months until release (*SD* = 13.52 months, range = 0–60.5 months). In total, young offenders had reported a mean life-time incarceration of 28.99 months (*SD* = 24.98 months, range = 0.27–147.53 months), with 58.5% being incarcerated one to 6 times before the index incarceration (*M* = 1.33, *SD* = 1.26). Only 25.5% (*n* = 27) had never been convicted before, whereas 74.5% (*n* = 79) reported at least one prior conviction, with *n* = 26 participants having been convicted once, *n* = 21 twice, and *n* = 32 at least three times before (*M* = 2.55, *SD* = 4.29, range = 0–30). Half of the sample (50.0%) had committed a violent offense before the index offense with one to 14 previous convictions for a violent offense (*M* = 1.75, *SD* = 2.72). Moreover, 48.1% of the participants had committed any delinquent acts even before reaching age of criminal responsibility.

Since we aimed at focusing on young offenders engaging in and desisting from SVC crime, we defined SVC offenders as proposed by previous research (18, 22): All participants who had been convicted at least 3 times before the index incarceration in which at least one of these convictions was based on a violent crime were considered SVC-pre offenders (others: non-SVC-pre offenders). All participants who were convicted at least 3 times after release from the index incarceration with at least one conviction for a violent crime were considered SVC-post offenders. All other offenders not reaching this threshold were considered SVC-post desisters.

### Measures

#### Mental Health

Young offenders' mental health was assessed using the Youth Self-Report (YSR)/Young Adult Self-Report (YASR) (23–26), which have been considered as the most widely used self-report scales for psychological/behavioral problems in young people [e.g., (27)]. A total of 112 (YSR) and 119 items (YASR)–each of them being scored from 0 (not true) to 2 (very true or often true) –can be assigned to 8 syndrome scales that build up to two higher-order problem scales: (1) the internalizing problem scale (“anxious/depressed,” “withdrawn,” “somatic complaints”), and (2) the externalizing problem scale (“aggressive behavior,” “rule-breaking behavior”). The syndrome scales “social problems,” “thought problems,” and “attention problems” are not assigned to any higher-order problem scale but are included in the total problem score. Raw values were transformed into standardized T-scores. Cut-offs indicating clinical significance of reported syndrome and problem scores are provided by the YSR/YASR manuals.

#### Substance Use

Young offenders' substance use was assessed in terms of alcohol and illegal drug consumption. Alcohol drinking behavior (frequency) and subsequent problems were examined by the German 10 item version of the Alcohol Use Disorders Identification Test [AUDIT (28–30)]. According to a participant's self-ratings, items can be scores from 0 to 4 points. A score of at least 8 points indicates alarming drinking habits. Illegal drug use was asked by means of the Structured Clinical Interview for DSM-IV [SCID-I (31)], which determines drug related problems in terms of dependence and abuse according to the DSM-IV criteria.

#### Personality Disorders

Personality disorders were assessed using to the ICD-10 international personality disorder examination [IPDE (32)], a semi-structured interview to consider personality disorders according to ICD-10 criteria as absent, probable or definite. For the present study, we used a binary coding with 1 (= probable/definite) and 0 (= no personality disorder).

#### Criminal Careers

As mentioned above, young offenders' criminal careers were analyzed using their official criminal records provided by the German Federal Office of Justice. Records were obtained in 2016, allowing a mean follow-up period of up to 15 years after release from the index incarceration (*M* = 156.90 months, *SD* = 14.07 months, range = 110.50–176.00 months). In Germany, criminal records contain information on any criminal conviction and incarceration but not criminal charges. For the present study, we were interested in whether or not participants had been convicted for any crime or violent crime in particular, and whether or not they had been incarcerated before and after release from the index incarceration.

### Statistical Analyses

Statistical analyses were performed in IBM SPSS Statistics Version 28 for Windows. Distributional differences among groups were analyzed by Chi^2^-tests. (M) ANOVAS, and t-tests. For the Chi^2^-tests, we considered the effect size Cramer's *V*, which portrays the strength of the association between two dichotomous variables. A Cramer's *V* larger than 0.25 is usually considered very strong, larger than 0.15 strong, larger than 0.10 moderate, and below 0.10 weak or very weak (33). Moreover, adjusted residuals (*AR*) indicate significant deviations from expected cell distributions with *AR* ≤ −2.0 or *AR* ≥ 2.0. Partial eta^2^ is a common effect size measure used in (M) ANOVA which reflects the proportion of variance associated with each main and interaction effect in the sample. It ranges from 0 and 1 and can be interpreted by using a rule of thumb (34), whereas a partial eta^2^ of .01 is considered as a small effect, of .06 a medium effect, and >0.14 a large effect. Further, Cohen's *d* was used as measure of effect size to indicate standardized differences between two means, whereby a Cohen's *d* of 0.01 is defined as very small, of 0.20 as small, of 0.50 as medium, of 0.80 as large, of 1.20 as very large and 2.0 as huge. Effect sizes bigger than 1 means that the difference between the two means is larger than one standard deviation, larger than 2 means larger the two standard deviations and so forth. Associations among variables (e.g., mental health and duration of incarceration) were examined by Pearson *r* correlations, which can vary between −1, a perfect negative correlation, to +1, a perfect positive correlation. According to Cohen (35, 36), this effect size is considered small if *r* varies around 0.1, medium around 0.3 and large if *r* > 0.5. Predictive effects of mental health, substance use, personality disorder, and covariates on SVC-post status were analyzed by (multiple) binary regression models. Odds Ratios (*OR*) quantify the strength of the associations between indicator variable and outcome status, with *OR* = 1 indicating equal odds to belong to either SVC-post desister or offender group, *OR* > 1 indicating increased chance of belonging to the SVC-post desister group, and *OR* < 1 indicating increased risk of belonging to the SVC-post offender group. Considering the abovementioned assumptions about increasing age being protective against criminal risk (17), we first analyzed the predictive effect of the covariate age on SVC-post offender status. Further, the predictive effect of the examined variables that were to be found to distinguish between SVC-post offenders and SVC-post desisters were analyzed under statistical control of age.

## Results

### SVC Status

Based on the abovementioned criteria, 33.0% of the sample (*n* = 35) were SVC-pre offenders and 57.5% (*n* = 61) SVC-post offenders. Twenty-four SVC-pre offenders also became SVC-post offenders (68.8%), whereas 37 (52.1%) of the SVC-post offenders had not been SVC-pre offenders. Eleven (31.2%) of the SVC-pre offenders did not become SVC-post offenders and 34 (47.9%) young offenders did not hold any SVC status before and after incarceration, thus representing a total of 45 (42.5%) SVC-post desisters. SVC status until and after index incarceration was not significantly associated, Chi^2^(1) = 2.60, *p* = 0.107, *AR* = 1.6.

Compared to non-SVC-pre offenders, SVC-pre offenders had higher numbers of previous convictions and incarcerations (see Table 1). SVC-post offenders differed from SVC-post desisters in terms of a younger age at the index offense and age at baseline assessment as well as by having lower educational levels and by having shown delinquent behavior before criminal responsibility more often (see Table 2).

**Table 1:** Differences between SVC-pre offenders and non-SVC-pre offenders.

	**SVC-pre (N** **=** **35)**			**non-SVC-pre (N** **=** **71)**			
	***M***	***SD***	***n (%)***	***M***	***SD***	***n (%)***	***T (df)***	***p***	***d***	*****χ^2^(1)*****	***Cramer's V***	***AR***
**Covariates**												
Age at the index offense	18.52	1.67		18.23	1.83		−0.73 (86)	0.470	−0.16			
Age at baseline assessment	19.80	3.37		19.38	1.96		−0.97 (104)	0.336	−0.20			
**Delinquency**												
Number of previous convictions	**4.63**	**5.97**		**1.52**	**2.67**		**−2.94** **(40.83)**	**0.003**	**−0.77**			
Number of previous incarcerations	**1.75**	**1.48**		**1.12**	**1.09**		**−2.26** **(84)**	**0.026**	**−0.52**			
Delinquent behavior before criminal responsibility			20 (57.1)			31 (43.7)		0.191		1.71	0.127	1.3
Lower educational level			22 (62.9)			34 (47.9)		0.147		2.11	0.141	1.5
Follow-up (months)	158.83	11.52		155.90	15.24		0.88 (77)	0.384	−0.21			
Any future conviction			31 (88.6)			58 (81.7)		0.364		0.82	0.088	0.9
Number of future convictions	40.71	103.60		13.61	17.06		−1.54 (34.91)	0.067	−0.45			
Future violent offenses			26 (74.3)			41 (57.7)		0.097		2.76	0.161	1.7
Number of future violent crimes	4.03	5.24		2.42	3.29		−1.66 (47.66)	0.052	−0.40			
Future incarcerations			**31** **(88.6)**			**49** **(69.0)**		**0.028**		**4.84**	**0.214**	**2.2**
Number of future incarcerations	**4.46**	**3.07**		**3.04**	**3.05**		**2.24** **(104)**	**0.027**	**−0.46**			
**Mental health**												
*Y(A)SR clinical cut-off exceeded*												
Social withdrawal			1 (2.9)			3 (4.2)		0.729		0.12	0.034	0.3
Somatic complaints			4 (11.4)			9 (12.7)		0.854		0.03	0.018	0.2
Anxious/depressed			4 (11.4)			7 (9.9)		0.802		0.06	0.024	0.0
Social problems			3 (8.6)			2 (2.8)		0.189		1.73	0.128	1.3
Thought problems			12 (34.3)			16 (22.5)		0.197		1.67	0.125	1.3
Attention problems			3 (8.6)			9 (12.7)		0.531		0.39	0.061	0.6
Rule-breaking behavior			19 (54.3)			31 (43.7)		0.303		1.06	0.100	1.0
Aggressive behavior			6 (17.1)			12 (16.9)		0.975		0.00	0.003	0.0
Internalizing problems			6 (17.1)			17 (23.9)		0.424		0.64	0.078	0.8
Externalizing problems			24 (68.6)			44 (62.0)		0.505		0.44	0.065	0.7
Total problem score			15 (42.9)			35 (49.3)		0.532		0.39	0.061	0.6
*SCID-I illegal drug use*			27 (77.1)			46 (64.8)		0.196		1.67	0.125	1.3
*AUDIT ≥ 8*			25 (71.4)			40 (56.3)		0.134		2.25	0.146	1.5
*IPDE personality disorder*												
Any			15 (42.9)			32 (45.1)		0.829		0.05	0.021	0.2
Cluster B			15 (42.9)			27 (38.0)		0.633		3.23	0.046	0.5
Anti-social			9 (25.7)			22 (31.0)		0.575		0.32	0.055	0.6
Emotionally-instable			10 (28.6)			17 (23.9)		0.601		0.26	0.050	0.5
Paranoid			2 (5.7)			5 (7.0%)		0.796		0.07	0.025	0.3
Schizoid			0 (0.0)			6 (8.5)		0.077		3.14	0.172	1.8
Histrionic			0 (0.0)			1 (1.4)		0.481		0.50	0.069	0.7
Obsessive			0 (0.0)			1 (1.4)		0.481		0.50	0.069	0.7
Anxious			0 (0.0)			1 (1.4)		0.481		0.50	0.069	0.7
Dependent			0 (0.0)			2 (2.8)		0.316		1.01	0.097	1.0

*Significant differences (p ≤ 0.05) are in bold*.

**Table 2:** Differences between SVC-post offenders and SVC-post desisters.

	**SVC-post (*****N*** **=** **61)**			**SVC-post desisters (*****N*** **=** **45)**			
	***M***	***SD***	***n (%)***	***M***	***SD***	***n (%)***	***T (df)***	***p***	***d***	*****χ**^2^(1)***	***Cramer's V***	***AR***
**Covariates**												
Age at the index offense	**18.06**	**1.89**		**18.93**	**1.30**		**2.47 (70.87)**	**0.035**	**0.50**			
Age at baseline assessment	**19.13**	**1.88**		**20.04**	**2.29**		**2.25 (104)**	**0.026**	**0.44**			
**Delinquency**												
Number of previous convictions	2.59	3.95		2.49	4.77		−0.12 (104)	0.905	−0.02			
Number of previous incarcerations	1.35	1.31		1.31	1.16		−0.14 (84)	0.887	−0.03			
Delinquent behavior before criminal responsibility			**35** **(57.4)**			**16** **(35.6)**		**0.026**		**4.94**	**0.216**	**2.2**
Lower educational level			**39** **(63.9)**			**17** **(37.8)**		**0.008**		**7.11**	**0.259**	**2.7**
Follow-up (months)	155.95	14.58		158.96	12.24		0.88 (77)	0.380	0.21			
Any future conviction		**61** **(100)**				**28** **(62.2)**		**<0.001**		**27.45**	**0.509**	**5.2**
Number of future convictions	**35.33**	**79.00**		**5.24**	**10.24**		**−2.94 (62.75)**	**0.004**	**−0.50**			
Future violent offenses			**61** **(100)**			**6** **(9.0)**		**<0.001**		**83.64**	**0.888**	**9.1**
Number of future violent crimes	**5.02**	**4.34**		**0.16**	**0.42**		**−8.68** **(61.55)**	**<0.001**	**1.47**			
Future incarcerations			**60** **(98.4)**			**20** **(44.4)**		**<0.001**		**40.67**	**0.619**	**6.4**
Number of future incarcerations	**5.07**	**2.72**		**1.40**	**2.27**		**−7.55** **(102.34)**	**<0.001**	**−1.44**			
**Mental health**												
*Y(A)SR clinical cut-off exceeded*												
Social withdrawal			4 (6.6)			0 (0.0)		0.080		3.07	0.170	1.8
Somatic complaints			8 (13.1)			5 (11.1)		0.756		0.10	0.030	0.3
Anxious/depressed			8 (13.1)			3 (6.7)		0.282		1.16	0.105	1.1
Social problems			4 (6.6)			1 (2.2)		0.298		1.08	0.101	1.0
Thought problems			18 (29.5)			10 (22.2)		0.400		0.71	0.082	0.8
Attention problems			9 (14.8)			3 (6.7)		0.194		1.69	0.126	1.3
Rule-breaking behavior			33 (54.1)			17 (37.8)		0.096		2.77	0.162	1.7
Aggressive behavior			10 (16.4)			8 (17.8)		0.851		0.04	0.018	0.2
Internalizing problems			**18 (29.5)**			**5 (11.1)**		**0.023**		**5.16**	**0.221**	**2.3**
Externalizing problems			43 (70.5)			25 (55.6)		0.113		2.51	0.154	1.6
Total problem score			33 (54.1)			17 (37.8)		0.096		2.77	0.162	1.7
*SCID-I illegal drug use*			46 (75.4)			27 (60.0)		0.090		2.87	0.164	1.7
*AUDIT ≥ 8*			**45 (73.8)**			**20 (44.4)**		**0.002**		**9.39**	**0.298**	**3.1**
*IPDE personality disorder*												
Any			**32 (52.5)**			**15 (33.3)**		**0.050**		**3.84**	**0.190**	**2.0**
Cluster B			**30 (49.2)**			**12 (26.7)**		**0.019**		**5.49**	**0.228**	**2.3**
Anti-social			21 (34.4)			10 (22.2)		0.172		1.86	0.133	1.4
Emotionally-instable			18 (29.5)			9 (20.0)		0.267		1.23	0.108	1.1
Paranoid			3 (4.9)			4 (8.9)		0.416		0.66	0.079	0.8
Schizoid			4 (6.6)			2 (4.4)		0.642		0.22	0.045	0.5
Histrionic			1 (1.6)			0 (0.0)		0.388		0.75	0.084	0.9
Obsessive			1 (1.6)			0 (0.0)		0.388		0.75	0.084	0.9
Anxious			1 (1.6)			0 (0.0)		0.388		0.75	0.084	0.9
Dependent			1 (1.6)			1 (2.2)		0.827		0.05	0.021	0.2

*Significant differences (p ≤ 0.05) are in bold*.

### Criminal Recidivism

As shown in Table 2, follow-up periods did not differ significantly between SVC-post offenders and SVC-post desisters. However, not only were SVC-post offenders more likely to show any further conviction, but they also had higher numbers of future convictions. Similar patterns were found concerning future violent offenses and future incarcerations.

### Mental Health, Substance Use, and Personality Disorders

**Figure 1:**
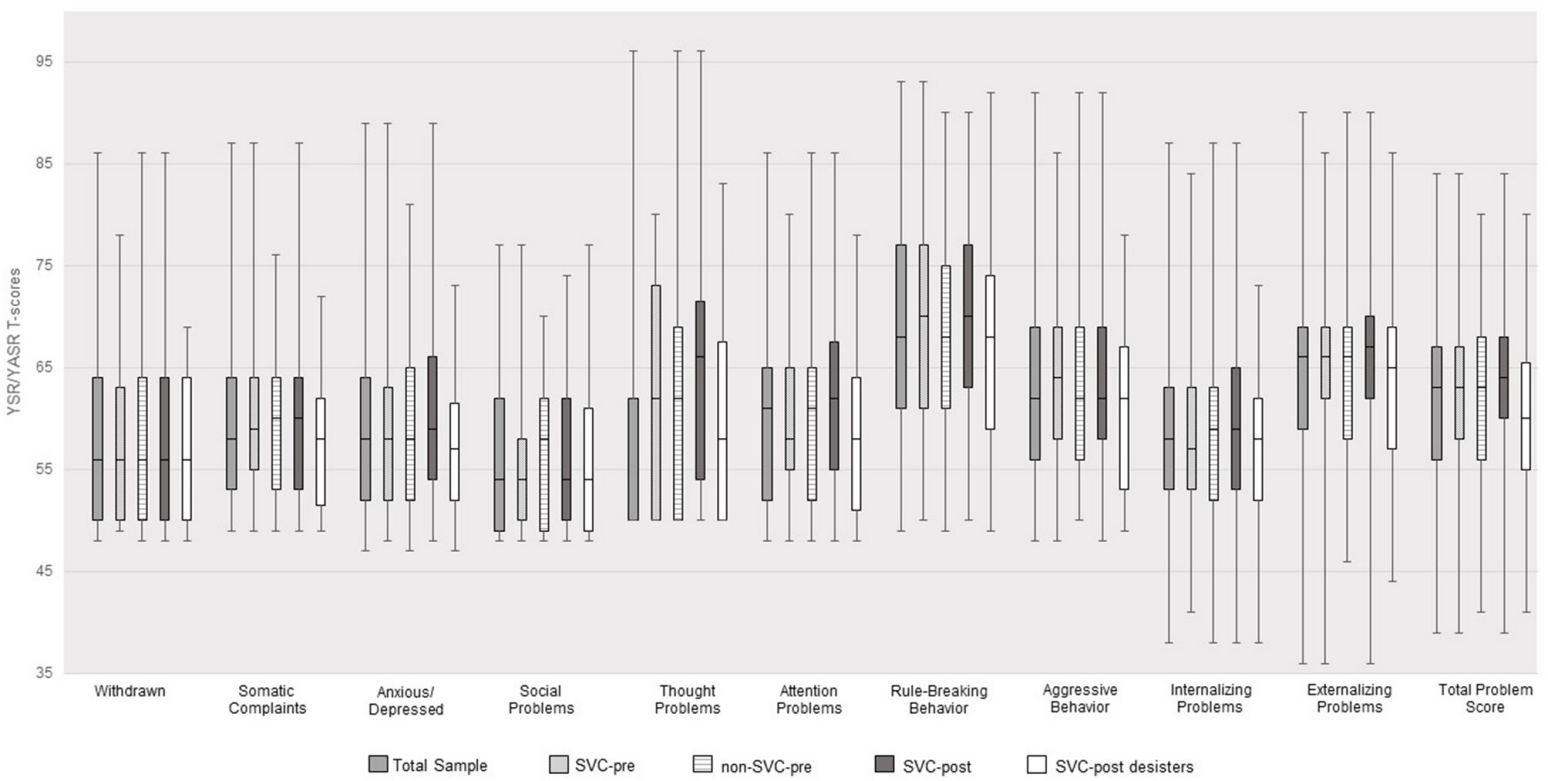
YSR/YASR T-Scores for the total sample (N = 106) and for SVC offender status.

T-scores (boxplots) on YSR/YASR scales for the total sample and dependent on SVC offender status are displayed in Figure 1. Overall, young offenders' scores fell close or into the borderline/clinical ranges as proposed by the YSR/YASR manuals. Scores on the anxious/depressed scale (*r* = −0.260, *p* = 0.007), the rule-breaking behavior scale (*r* = −0.208, *p* = 0.033), and the internalizing problems scale (*r* = −0.193, *p* = 0.048) were negatively associated with index incarceration time until study assessment. However, scores on withdrawn (*r* = 0.299, *p* = 0.007) and internalizing problems (*r* = 0.244, *p* = 0.030) were positively associated with remaining time to release. Although no significant differences emerged between SVC-pre and non-SVC-pre offenders, SVC-post offenders showed significantly higher scores than SVC-post desisters regarding attention problems, *F* (1, 104) = 5.05, *p* = 0.027, partial eta^2^ = 0.05, and total problems, *F* (1, 104) = 4.41, *p* = 0.038, partial eta^2^ = 0.04.

**Figure 2:**
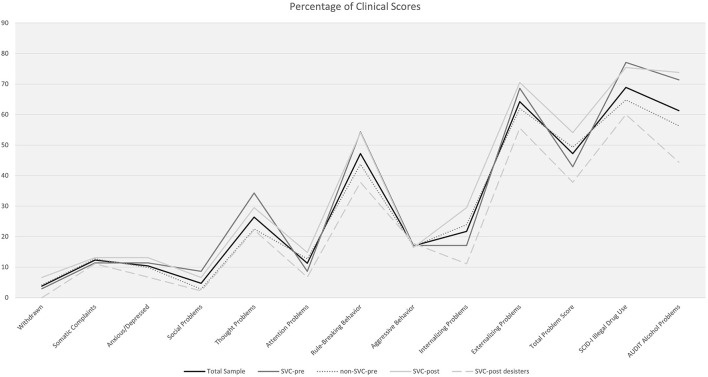
Percentages of YSR/YASR, SCID-I and AUDIT clinical scores for the total sample (*N* = 106) and for SVC offender status.

Figure 2 as well as Tables 1, 2 show the percentages of participants exceeding clinical cut-offs on the YSR/YASR scales, AUDIT, and SCID-I substance use problems. SVC-post offenders more often exceeded clinical cut-offs regarding internalizing problems and alarming alcohol use compared to SVC-post desisters (see Table 2). When summing up clinically relevant problem scales (min. = 0, max. = 10), more than 75% of the total sample showed at least a sum score of 2 (*M* = 2.63, *SD* = 1.73, range = 0–8). No differences emerged between SVC-pre and non-SVC-pre offenders, whereas SVC-post offenders (*M* = 3.03, *SD* = 1.71) showed higher burden than SVC-post desisters (*M* = 2.09, *SD* = 1.63), *t* (104) = 2.86, *p* = 0.005, *d* = −0.56).

Personality disorders were probable/definite in 44.3% (*n* = 47) of the total sample, with cluster B personality disorders being most prevalent (*n* = 42, 39.6%). Antisocial personality disorder was found in 29.2% (*n* = 31) and emotionally unstable personality disorder in 25.5% (*n* = 27) of the participants. Further personality disorders found in the present sample were paranoid (*n* = 7, 6.6%), schizoid (*n* = 6, 5.7%, dependent (*n* = 2, 1.9%), histrionic, obsessive, and anxious (each *n* = 1, 0.9%) personality disorder. Whereas most of the young offenders were not probable of having a personality disorder (*n* = 59, 55.7%), more than one fourth was assigned to one (*n* = 28, 26.4%), 13 (12.3%) two, and 6 (17.0%) to more than three personality disorders (*M* = 0.72, *SD* = 1.02, range = 0–5). No differences emerged between SVC-pre and non-SVC-pre offenders in the distribution of personality disorders (see Table 1). However, SVC-post offenders more often showed any - especially cluster B - personality disorder compared to SVC-post desisters (see Table 2).

### Prediction of SVC Desistance

With regard to abovementioned assumptions about reduced criminal risk with increasing age (13), we first analyzed the predictive effect of the covariate age on SVC-post offender status. The binary regression model indicated that increasing age was positively associated with the chance of being a SVC-post desister (*OR* = 1.25, 95%*CI* = 1.02–1.53, *p* = 0.032). Second, we analyzed single predictive effects of those variables that had been found to distinguish between SVC-post offenders and SVC-post desisters under statistical control of age. As shown in Table 3, lower educational level, clinically relevant mental health problems, alarming alcohol use, higher number of personality disorders and, especially, presence of cluster B personality disorder were negatively associated with the chance of SVC desistance. Third, when all these predictors were considered simultaneously, only alcohol use remained significantly associated with SVC-post offender status (*OR* = 0.36, 95%*CI* = 0.13–0.96, *p* = 0.042).

**Table 3:** Binary regression analyses on SVC-post offender status (single predictors).

**Independent variables**	**SVC-post desistance**	
	**OR**	**95% CI**
Delinquency before criminal responsibility (cat.)	2.21	0.98–4.96
Low education (cat.)	0.42^*^	0.18–0.99
YSR attention problems (dim.)	0.96	0.91–1.02
YSR internalizing problems (cat.)	0.36	0.12–1.10
YSR total problem score (dim.)	0.96	0.92–1.01
YSR total problem score (cat.)	0.41^*^	0.18–0.98
Alcohol problems (cat.)	0.32^**^	0.14–0.75
Sum of personality disorders (dim.)	0.79^*^	9.63–1.00
Any personality disorder (cat.)	0.49	0.22–1.10
Cluster B personality disorder (cat.)	0.42^*^	0.18–0.98

*OR, odds ratio; CI, confidence interval; Cat, categorical; dim., dimensional. Analyses controlled for age*.

* *p ≤ 0.05*,

** *p ≤ 0.01*.

## Discussion

Mental health problems are common among young offender samples but their role in predicting criminal recidivism is still not clear. Early identification and treatment of young offenders at risk of SVC offending is of major importance to increase their chances to develop into a healthy and non-criminal future and protect society from further crime. The present study aimed at contributing to and expanding the current knowledge on the dynamics of mental health and SVC offending by examining mental health status and long-term courses of delinquency in a high-risk sample of young detainees.

Consistent with previous research [e.g., (1)], we found a high prevalence of mental health issues in the present sample, especially in terms of externalizing problems. Internalizing behaviors including problems with anxiety and depression as well as rule-breaking behaviors appeared to be higher with shorter incarceration time until assessment, whereas social withdrawal and internalizing problems increased with longer time remaining until release. Although effects were rather small, they might reflect a particular dependency of mental health issues on incarceration time, especially at the beginning when young offenders need to adapt to the circumstances of incarceration, but also when facing rather long-lasting imprisonment. Whereas the initial phase of incarceration may thus be associated with feelings of loneliness, fear and uncertainty on the one hand and rule-breaking, oppositional behavior on the other hand, extended imprisonment may evoke thoughts and feelings of hopelessness and pointlessness in young offenders (37). These findings emphasize the need of an adequate monitoring of young detainees' mental health not only at the beginning but over the course of incarceration, especially in those facing long-term imprisonment.

One third of our sample met the criteria of being a SVC offender until assessment but more than half of the young offenders were identified as SVC offenders after release. Compared to previous studies (17, 18), SVC offending prevalence was rather high, which may be due to the fact that we focused on a high-risk incarcerated sample instead of somewhat broader and more heterogeneous juvenile justice samples. Although about 68% of the young offenders who identified as SVC offenders before assessment also showed SVC offending after release, SVC-status before assessment and after release were not significantly associated. This finding is not consistent with our initial hypothesis but suggests that also young offenders with a history of severe offending may still be able to desist from SVC offending. On the other hand, more than half of the SVC-post offenders had not shown SVC offending before, highlighting the need of effective early identification to reduce young people's risk of engaging in serious, violent, chronic delinquent careers.

Early identification is challenging due to the multifactorial etiology of criminal behavior. In the present study, differences between young people with SVC offending before assessment and those without were only found in terms of their prior criminal involvement, with SVC-pre offenders showing higher rates of previous convictions and incarcerations, which was expected based on the definition of SVC offending. More interestingly, no differences emerged regarding mental health. However, young offenders without SVC offending after release differed from those with SVC-post offending in several ways. First, SVC desisters were less likely to have shown early involvement in crime (i.e., delinquency before the age of criminal responsibility) and held higher academic qualifications. These findings corroborate previous research that pointed to more disadvantageous social conditions in young individuals engaging in continuous and severe criminal conduct (17). Early onset of criminal behavior and low academic achievement may both display indicators for deficient social integration and control early in life, thus highlighting the need to implement adequate support offers, e.g., in terms of youth welfare measures or family-based treatment approaches such as multisystemic therapy (38). Regarding mental health, SVC desisters reported fewer mental health problems in general and especially fewer externalizing behaviors, attention problems, alarming alcohol use, and personality disorders (cluster B personality disorders in particular). Statistically controlling for the influence of age, higher level of school education, less mental health issues as well as absence of alarming alcohol use and absence of cluster-B personality disorders predicted desistance from SVC offending in univariate analyses, although solely alcohol consumption remained a significant predictor in multiple regression. These findings are in line with previous research stating that criminal recidivism in young offenders is associated with mental health issues, substance use problems, and cluster-B personality disorders [e.g., (2, 9, 10)]. Substance use problems were found to be associated with increased risk of violence perpetration and to predict future violent and also SVC offending (8, 17, 39). However, the present results stress that not substance use problems in general may contribute to increased risk of future SVC offending, but alarming alcohol consumption in particular. Similar findings with regard to future violent offending were reported by previous research [e.g., (10)]. Alcohol use problems among young offenders are concerning in different ways. First, research has repeatedly emphasized that alcohol can affect an individual's emotional and behavioral regulation capacity and lower the threshold to engage in aggressive and violent acts. A recent meta-analysis stated a causal relationship between alcohol (but not stimulant drug) intoxication and aggression (40). Parrot and Eckhardt (41) introduced the alcohol-aggression link within I3 [e.g., (42)] and Alcohol Myopia Theory (43). According to the authors, I3 theory stresses that behavior is influenced by instigating, impelling, and inhibitory factors. Aggressive behavior may, thus, be probable when self-regulation is inhibited by the influence of alcohol in case a person is provoked and does show traits or attitudes in favor of aggressive (violent) behavior. Alcohol Myopia Theory highlights distorted attention processes due to alcohol influences with focus on short-term situational goals (e.g., lowering frustration) while neglecting long-term (legal) consequences. Second, alcohol is easily accessible, in Germany even legally as early as at the age of 16 years. The availability of and easy access to alcohol may contribute to the development of problematic alcohol use patterns, especially in those young people who suffer from early psychosocial burden and societal problems. Thus, prevention and intervention approaches addressing alcohol use in young people appear beneficial in order to prevent further dysfunctional outcomes, e.g., in terms of continuous criminal careers [e.g., (44)].

The interpretation of the present results requires the consideration of several strengths and limitations. First, we assessed a multitude of indicators for mental health including internalizing and externalizing problems, personality disorders, and alcohol and other (illegal) substance use problems. We combined both self-rated and clinician-administered measures and relied on well-established instruments. The long-term observation of criminal careers up to 15 years after release from incarceration allowed a more sophisticated insight into pathways of criminal offending beyond adolescence, which is of major importance in light of age-dependent crime prevalence [e.g., (5, 13)]. In the same regard, focus on continuous SVC offending is crucial to identify those young individuals who are in greatest need of prevention and treatment in order to reduce maladaptive personal but also societal consequences. On the other hand, the present sample represented a high-risk sample of young detainees, thus generalization to and implication for somewhat more heterogeneous juvenile offender samples is limited. Moreover, although sample size appeared satisfactory for long-term forensic examination, it was still rather small compared to general population studies. Because our sample size was predetermined by data availability, we did not perform a priori power calculations. However, post-hoc power analyses have been criticized as well (45). Yet, we conducted sensitivity analyses in G^*^Power Version 3.1.9.7 (46) that indicated, for example, that group differences between post-SVC offenders and desisters would have required at least an effect of *d* = 0.55 to be detected with a power of.80. Thus, the limited sample size (and statistical power) available in the present study may bear the risk of leaving some more subtle effects undetected due to statistical insignificance. Similarly, future research may benefit from examining female offenders, too, because gender influences have been discussed both in the dynamics between mental health and criminal recidivism as well as in the field of SVC offending (2, 16, 47). Second, although self-reports of mental health issues have been used in offender samples before, there is a risk of biased estimates due to under- or over-reporting [e.g., (27)]. Besides, there could be a possible bias in the self-rating instruments, as participants could have answered in a socially desirable manner, which is common in different settings, however, especially in offender samples. Likewise, despite the common scientific procedure in forensic psychology and psychiatry research of relying on officially registered crime, not all offenses may come to the attention of law enforcement agencies. Eventually, the consideration of other influencing factors underlying the effects of mental health on criminal behavior was beyond the scope of the present study. For instance, a vast amount of research has focused on ACEs as potential exploratory factors in the context of mental health and adolescent and adult (SVC) criminal behavior (9, 18, 22, 27, 48). More research is needed to broaden the knowledge on the associations between maladaptive developmental factors including mental health and perpetration but also desistance from criminal behavior in order to derive early and effective prevention and treatment approaches aimed at reducing young people's risk of engaging in continuous (SVC) criminal careers and, thus, support their development into a healthy, non-delinquent future.

## Data Availability Statement

The datasets presented in this article are not readily available because of the specific confidentiality of the assessed clinical and forensic information. Scientists wishing to use them for non-commercial purposes are kindly asked to contact the present authors in order to frame individual agreements. Requests to access the datasets should be directed to steffen.barra@uks.eu.

## Ethics Statement

The studies involving human participants were reviewed and approved by Ethics Committee of the Medical Chamber of Saarland, Saarbrücken, Germany. Written informed consent to participate in this study was provided by the participants themselves in case they were at least 18 years old at time of assessment or participants' legal guardian when younger.

## Author Contributions

SB, WR, DT, and MR: conceptualization. SB and WR: methodology and data curation. SB: formal analysis, writing—original draft preparation, and visualization. WR, MR, and PR-J: investigation. WR and MR: resources and project administration. SB, WR, DT, PR-J, and PH: writing—review and editing. All authors have read and agreed to the published version of the manuscript.

## Funding

We acknowledge support by the Deutsche Forschungsgemeinschaft (DFG, German Research Foundation) and Saarland University within the Open Access Publication Funding programme.

## Conflict of Interest

The authors declare that the research was conducted in the absence of any commercial or financial relationships that could be construed as a potential conflict of interest.

## Publisher's Note

All claims expressed in this article are solely those of the authors and do not necessarily represent those of their affiliated organizations, or those of the publisher, the editors and the reviewers. Any product that may be evaluated in this article, or claim that may be made by its manufacturer, is not guaranteed or endorsed by the publisher.
